# Supplementary material for: Menstrual Hygiene Preparedness Among Schools in India: A Systematic Review and Meta-Analysis of System-and Policy-Level Actions
Source: Int J Environ Res Public Health. 2020 Jan 19;17(2):647. doi: 10.3390/ijerph17020647 (PMC7013590; doi:10.3390/ijerph17020647)
Supplement: Supplementary file 1 [file ijerph-17-00647-s001.zip › Final Quality assessment sheet MHM meta analysis.rtf]

Supplementary Table S1. Description of the qualitative assessment of the studies included in the review (n=133)
Firth Author	Year  of publication	Sample size*	Randomness
of sample**	Inclusion & exclusion Criteria†	Details of enrolled and analyzed‡	Completeness of the outcomes¶	Participants' characteristics§	Details of statistical analysis§§	Total score (Max=7)	
Gupta J, et al.	2001	N	N	N	Y	Y	Y	N	3	
Mahajan P, et al.	2004	N	Y	N	N	Y	N	Y	3	
Deo DS, et al	2005	N	N	N	N	N	N	N	0	
Khanna A, et al.	2005	N	Y	N	Y	N	Y	N	3	
Gupta S, et al.	2006	N	N	N	Y	N	N	N	1	
Tiwari H, et al.	2006	Y	Y	N	Y	Y	N	N	4	
Dasgupta A, et al.	2008	N	N	N	N	N	Y	Y	2	
Kotecha PV, et al.	2009	N	Y	N	N	Y	Y	Y	4	
Mittal K, et al.	2010	Y	Y	N	N	N	N	N	2	
Mudey AB, et al.	2010	N	N	N	N	Y 	N	N	1	
Omidvar S, et al.	2010	N	N	N	N	N	Y	Y	2	
Dorle AS, et al.	2010	Y	Y	N	N	N	N	N	2	
Verma PB, et al.	2011	N	N	N	N	Y	N	N	1	
Thakre SB, et al.	2011	N	N	N	N	Y	Y	N	2	
Venkatesh R, et al.	2011	N	N	N	N	N	N	N	0	
Dube S, et al.	2012	N	Y	N	N	Y	N	N	2	
Jothy K, et al.	2012	N	Y	N	N	Y	Y	N	3	
Kamaljit K, et al.	2012	N	N	N	N	Y	Y	N	2	
Shanbhag D, et al.	2012	N	N	N	N	Y	Y	N	2	
Sudeshna R, et al.	2012	Y	N	N	Y	Y	Y	Y	5	
Datta A, et al.	2012	N	N	N	N	Y	Y	Y	3	
Khan A.	2012	N	Y	Y	N	N	N	N	2	
Solanki H, et al.	2012	N	N	N	N	Y	N	N	1	
Dambhare DG, et al.	2012	N	Y	N	Y	Y	N	N	3	
Salve SB, et al.	2012	N	N	N	N	Y	N	N	1	
Nair MKC, et al.	2012	Y	Y	Y	N	Y	Y	Y	6	
Verma P, et al.	2013	N	N	N	N	Y	Y	N	2	
Yasmin S, et al.	2013	N	Y	N	N	Y	Y	N	3	
Ade A, et al.	2013	N	N	N	N	N	Y	N	1	
Bhattacherjee S, et al.	2013	Y	Y	N	Y	Y	Y	Y	6	
Juyal R, et al.	2013	Y	Y	N	Y	N	N	N	3	
Kanotra SK, et al.	2013	N	N	N	N	N	N	N	7	
Kumar D, et al.	2013	Y	Y	Y	N	Y	Y	N	5	
Kamath R, et al.	2013	Y	Y	N	N	Y	Y	N	4	
Amirtha G, et al.	2013	N	N	N	N	N	N	N	0	
Arora A, et al.	2013	N	Y	N	Y	N	N	N	2	
Katiyar K, et al.	2013	N	Y	N	N	Y	Y	N	3	
Paria B, et al.	2014	N	N	N	N	Y	Y	N	2	
Katkuri S, et al.	2014	N	N	N	N	Y	N	N	1	
Nagamani NG, et al.	2014	N	N	N	N	Y	N	N	1	
Raina D, et al.	2014	N	N	N	N	Y	N	N	1	
Patle R, et al.	2014	Y	N	N	N	Y	N	N	2	
Pandit D, et al.	2014	N	Y	N	Y	Y	Y	Y	5	
Thakur H, et al.	2014	N	Y	Y	Y	Y	Y	Y	6	
Patavegar BN, et al.	2014	Y	Y	Y	N	Y	Y	N	5	
Anusree PC, et al.	2014	N	N	N	N	Y	Y	Y	3	
Jailkhani SMK, et al.	2014	N	N	N	N	Y	N	N	1	
Sowrnya BC, et al.	2014	N	N	N	N	Y	N	N	1	
Jain A, et al.	2014	N	N	N	N	Y	Y	N	2	
Madhusudan M, et al.	2014	N	N	N	N	Y	Y	Y	3	
Lalbiaknungi L, et al.	2015	N	N	N	N	Y	Y	N	2	
Prajapati D, et al.	2015	N	N	N	N	Y	Y	N	2	
Zaidi SHN, et al.	2015	N	N	Y	N	Y	Y	N	3	
Langer B, et al.	2015	N	N	N	N	N	N	Y	1	
Preeti G, et al.	2015	N	Y	N	N	N	N	N	1	
Anitha S, et al.	2015	N	N	N	N	N	N	N	0	
Varghese M, et al.	2015	N	Y	Y	N	Y	Y	Y	5	
Walia DK, et al.	2015	N	N	N	N	Y	N	N	1	
Tarhane S, et al.	2015	N	N	N	N	Y	N	N	1	
Mohanty S, et al.	2016	N	N	Y	N	Y	Y	Y	4	
Chadalawada UR, et al.	2016	N	N	N	N	Y	N	N	1	
Kansal S, et al.	2016	Y	Y	Y	Y	Y	Y	Y	7	
Seenivasan P, et al.	2016	Y	N	N	N	N	N	N	1	
Kshirsagar et al.	2016	N	N	N	N	N	N	N	0	
Kanchan C, et al.	2016	N	N	N	N	N	N	N	0	
Dudeja P, et al.	2016	Y	N	N	Y	N	N	N	2	
Anju CM, et al.	2016	N	N	N	N	Y	N	N	1	
Ramachandra K, et al.	2016	N	N	N	N	Y	N	N	1	
Devi U, et al.	2016	N	N	N	N	Y	N	N	1	
Taklikar C, et al.	2016	N	N	N	N	Y	Y	N	2	
Nagaraj C, et al.	2016	Y	N	Y	Y	Y	N	Y	5	
Maji S.	2016	N	N	N	N	Y	Y	N	2	
Chauhan A, et al.	2016	N	N	N	N	Y	N	N	1	
Malhotra A, et al.	2016	Y	Y	Y	Y	Y	Y	Y	7	
Singhal VK.	2016	Y	N	N	N	Y	N	N	2	
Sarvade A, et al.	2016	N	N	N	N	Y	N	N	1	
Sharma S, et al.	2016	N	N	N	N	Y	N	N	1	
Kusuma ML, et al.	2016	Y	Y	Y	N	Y	Y	Y	6	
Rokade HG, et al.	2016	Y	Y	Y	N	Y	Y	Y	6	
Bachloo T, et al.	2016	N	Y	Y	N	Y	Y	Y	5	
Jitpure S.	2016	N	N	N	N	Y	N	N	1	
Pal J, et al.	2017	N	N	N	N	N	Y	Y	2	
Darivemula, et al.	2017	Y	Y	N	N	N	N	N	2	
Sarkar I, et al.	2017	N	N	Y	N	Y	Y	Y	4	
Bedi R, et al.	2017	N	N	N	N	Y	Y	N	2	
Javalkar SR, et al.	2017	N	N	N	N	N	Y	Y	2	
Tuli AG, et al.	2017	N	N	N	N	Y	N	N	1	
Singh P, et al.	2017	N	N	N	N	N	N	N	0	
Hakim A, et al.	2017	N	N	Y	N	Y	N	N	2	
Agarwal V, et al.	2017	Y	Y	N	N	N	Y	N	3	
Mathiyalagen P, et al.	2017	Y	Y	Y	N	N	Y	Y	5	
Shoor P, et al.	2017	N	N	Y	Y	Y	N	Y	4	
Neelkanth N, et al.	2017	N	N	N	N	Y	N	N	1	
Naithani R, et al.	2017	N	N	N	N	Y	Y	N	2	
Vandana V, et al.	2017	N	N	Y	N	Y	Y	N	3	
Dabade KJ, et al.	2017	N	N	Y	N	Y	N	N	2	
Kapoor G, et al.	2017	N	N	N	N	Y	Y	Y	3	
Hema Priya S., et al.	2017	N	Y	Y	Y	Y	Y	Y	6	
Gandotra N, et al.	2018	N	N	Y	N	N	N	N	1	
Deshpande TN, et al.	2018	Y	N	N	N	N	Y	N	2	
Gupta N, et al.	2018	N	N	Y	N	Y	Y	N	3	
Agarwal N, et al.	2018	N	N	N	N	       Y	N	N	1	
Tarannum F, et al.	2018	Y	Y	Y	N	Y	Y	Y	6	
Vashisht A, et al.	2018	Y	Y	N	Y	Y	Y	Y	6	
Chauhan P, et al.	2018	N	N	Y	N	N	N	N	1	
Tiwari A, et al.	2018	N	N	N	N	Y	N	Y	2	
Kakeri M, et al.	2018	N	N	N	N	Y	N	N	1	
Dharni IT.	2018	N	N	Y	N	Y	Y	N	3	
Kavitha M, et al.	2018	Y	Y	N	N	Y	Y	N	4	
Senapathi P, et al.	2018	N	N	N	N	Y	Y	N	2	
Gupta P, et al.	2018	N	Y	N	N	Y	N	N	2	
Rastogi S, et al.	2019	N	Y	N	N	Y	N	Y	3	
Sivakami M, et al.	2019	Y	Y	Y	Y	Y	Y	Y	7	
Patel SM, et al.	2019	N	N	Y	N	Y	N	N	2	
Nath KR, et al.	2019	Y	Y	Y	N	Y	Y	Y	6	
Khatuja R, et al.	2019	N	N	N	N	Y	N	N	1	
Pradhan S, et al.	2019	N	N	Y	N	Y	Y	Y	4	
Sangra S, et al.	2019	Y	N	Y	N	Y	N	N	3	
Madhavi KVP, et al.	2019	Y	Y	Y	N	Y	N	N	4	
Chajlana SPS, et al.	2019	N	N	Y	N	Y	Y	N	3	
Budemelli S, et al.	2019	Y	Y	Y	Y	Y	Y	N	6	
Das N, et al.	2019	Y	Y	N	Y	Y	Y	Y	6	
Sonowal P, et al.	2019	Y	N	N	N	Y	Y	    N	3	
Jyothi B, et al.	2019	Y	Y	Y	N	Y	Y	Y	6	
Chaudhary N, et al.	2019	Y	N	N	N	Y	Y	N	3	
Kamboj N, et al.	2019	N	N	N	N	Y	N	N	1	
Gupta S, et al.	2019	N	N	N	N	Y	Y	N	2	
Parle J, et al.	2019	N	N	N	N	Y	Y	N	2	
Kaur S.	2019	N	N	N	N	N	N	N	0	
Solvig N, et al.	2019	N	N	N	N	Y	N	N	1	
Manuja LM, et al.	2019	N	N	Y	N	Y	Y	Y	4	
Vaishampayar N, et al.	2019	N	N	N	N	Y	N	N	1	
Kalyani S, et al.	2019	N	N	N	N	Y	N	N	1	
*Was sample size calculation done?
**Was sample randomly distributed?
†Were inclusion and exclusion criteria mentioned in the methodology section?
‡Did authors mention how many cases were approached, how many were enrolled and included in the analysis?
¶Was outcome data adequately mentioned or completely given?
§Did authors specify participants' sociodemographic characteristics adequately?
§§Did authors mention what statistical analysis were done and what associations were looked at in the methodology section adequately?
Y= Yes, which has been rated 1 whereas N=No, which has been rated 0 while calculating total score
